# Supplementary material for: Role of Inflammation and Redox Status on Doxorubicin-Induced Cardiotoxicity in Infant and Adult CD-1 Male Mice
Source: Biomolecules. 2021 Nov 19;11(11):1725. doi: 10.3390/biom11111725 (PMC8615472; doi:10.3390/biom11111725)
Supplement: Supplementary file 1 [file biomolecules-11-01725-s001.zip › biomolecules-1438043-supplementary.pdf]

Supplementary Materials

Article

Role of inflammation and redox status on doxorubicin-induced cardiotoxicity in infant and adult CD-1 male mice

Ana Reis-Mendes[1,2,*], Ana Isabel Padrão[3], José Alberto Duarte[3,4], Salomé Gonçalves-Monteiro[5], Margarida Duarte-Araújo[6], Fernando Remião[1,2], Félix Carvalho[1,2], Emília Sousa[7,8], Maria Lourdes Bastos[1,2] and Vera Marisa Costa[1,2,*]

[1] Associate Laboratory i4HB - Institute for Health and Bioeconomy, Laboratory of Toxicology, Department of Biological Sciences, Faculty of Pharmacy, University of Porto, 4050-313 Porto, Portugal; afreis.mendes@gmail.com (A.R.-M.); remiao@ff.up.pt (F.R.); felixdc@ff.up.pt (F.C.); mlbastos@ff.up.pt (M.L.B.); veramcosta@ff.up.pt (V.M.C.)

[2] UCIBIO - Applied Molecular Biosciences Unit, REQUIMTE, Laboratory of Toxicology, Department of Biological Sciences, Faculty of Pharmacy, University of Porto, 4050-313 Porto, Portugal

[3] Research Center in Physical Activity, Health and Leisure (CIAFEL), Laboratory for Integrative and Translational Research in Population Health (ITR), Faculty of Sport, University of Porto, 4200-450 Porto, Portugal; apadrao@fade.up.pt (A.I.P.); jarduarte@fade.up.pt (J.A.D.)

[4] TOXRUN – Toxicology Research Unit, University Institute of Health Sciences, Advanced Polytechnic and University Cooperative (CESPU), CRL, 4585-116 Gandra, Portugal

[5] Outcomes Research Lab, MOREHealth, Outcomes Research Lab, Portuguese Institute of Oncology at Porto Francisco Gentil (IPO Porto), 4200-072 Porto, Portugal; salomemonteiro8180@gmail.com (S.G.-M.)

[6] Department of Immuno-Physiology and Pharmacology, ICBAS - Institute of Biomedical Sciences Abel Salazar, University of Porto, 4050-313 Porto, Portugal; mdcma@icbas.up.pt (M.D.-A.)

[7] Laboratory of Organic and Pharmaceutical Chemistry, Chemistry Department, Faculty of Pharmacy, University of Porto, 4050-313 Porto, Portugal; esousa@ff.up.pt (E.S.)

[8] CIIMAR – Interdisciplinary Centre of Marine and Environmental Research, 4450-208 Porto, Portugal.

* Correspondence: afreis.mendes@gmail.com (A.R.-M.); veramcosta@ff.up.pt (V.M.C.); Tel.: +351-220-428-599 (V.M.C.)

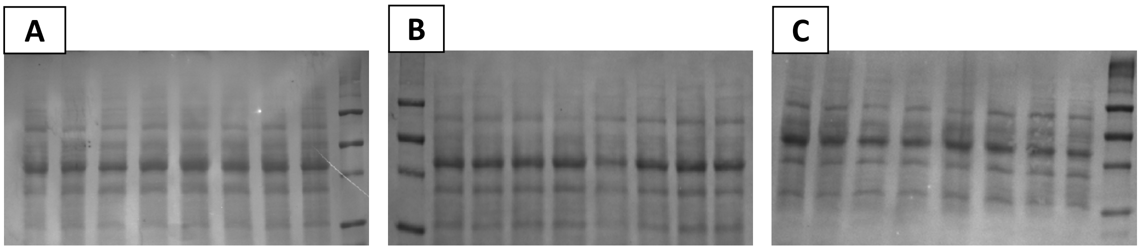

Figure S1. Loading control of Ponceau S staining of **(A, B, C)** nuclear factor erythroid-2 related factor 2 (Nrf2) (97 kDa) from infant and adult mice exposed to a cumulative dose of 18.0 mg/kg DOX or control animals.

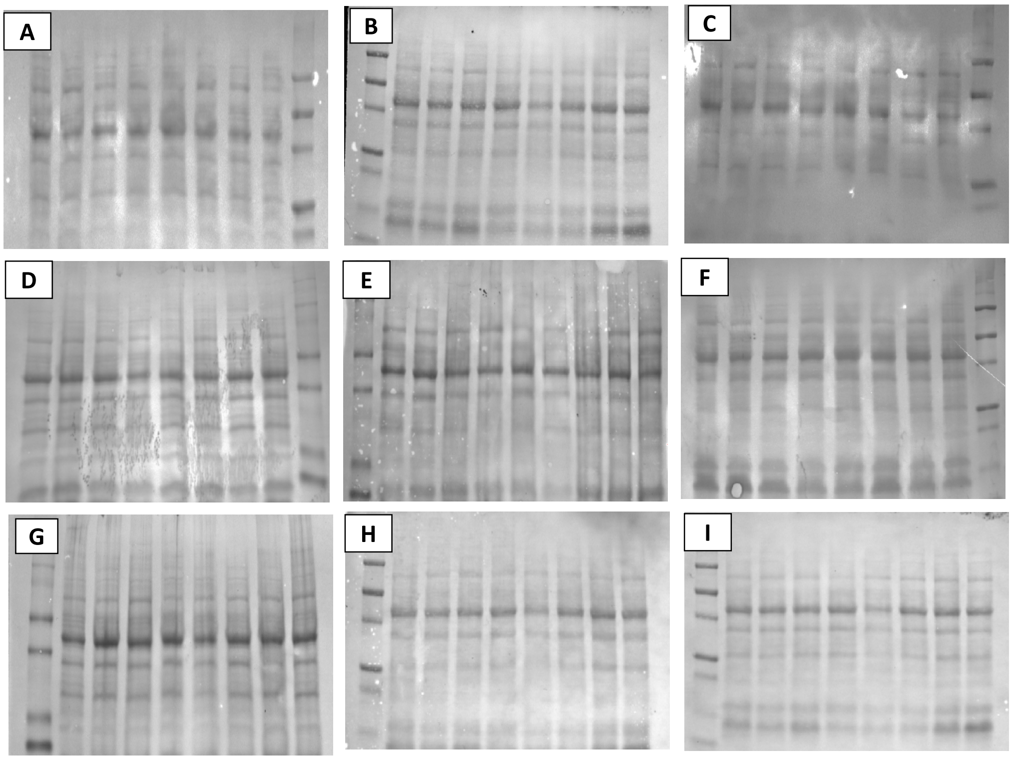

Figure S2. Loading control of Ponceau S staining of **(A, B, C)** p38 mitogen-activated protein kinase (p38 MAPK) (40 kDa), **(D, E, F)** nuclear factor kappa B (NF-κB) p65 (60 kDa) and NF-κB p52 (50 kDa), **(G, H, I)** myeloperoxidase (MPO) (48 kDa) from infant and adult mice exposed to a cumulative dose of 18.0 mg/kg DOX or control animals.

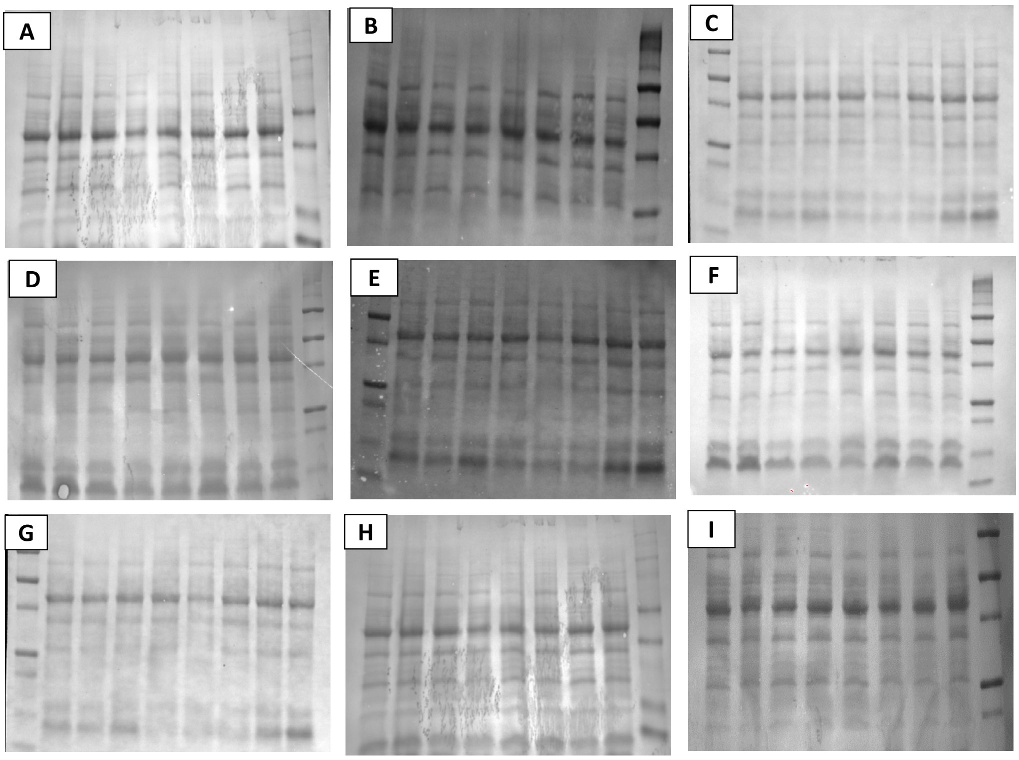

Figure S3. Loading control of Ponceau S staining of **(A, B, C)** Interleukin (IL)-1 beta (35 kDa), **(D, E, F)** IL-6 (24 kDa), **(F, G)** tumour necrosis factor-α (TNF-α) (25 kDa), **(H, I)** type 2 tumor necrosis factor receptor (TNFR2) (75 kDa) from infant and adult mice exposed to a cumulative dose of 18.0 mg/kg DOX or control animals.
